# From Balloon to Crystalline Structure in the Calcium Phosphate Flow-Driven Chemical Garden

**DOI:** 10.1021/acs.langmuir.3c00079

**Published:** 2023-03-27

**Authors:** Réka Zahorán, Pawan Kumar, Ágota Deák, Emese Lantos, Dezső Horváth, Ágota Tóth

**Affiliations:** † Department of Physical Chemistry and Materials Science, University of Szeged, Rerrich Béla tér 1, Szeged H-6720, Hungary; ‡ Department of Physical Chemistry and Materials Science, Interdisciplinary Excellence Centre, University of Szeged, Aradi sq. 1, Szeged 6720, Hungary; ¶ Department of Applied and Environmental Chemistry, University of Szeged, Rerrich Béla tér 1, Szeged H-6720, Hungary

## Abstract

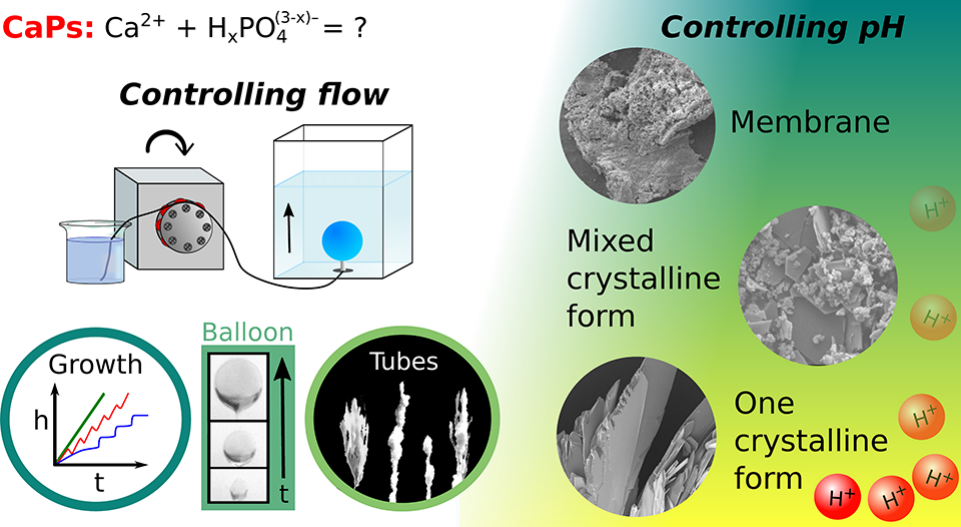

We have studied the calcium phosphate precipitation reaction by producing chemical gardens in a controlled manner using a three-dimensional flow-driven technique. The injection of the phosphate containing solution into the calcium ion reservoir has resulted in structures varying from membranes to crystals. Dynamical phase diagrams are constructed by varying chemical composition and flow rates from which three different growth mechanisms have been revealed. The microstructural analysis by scanning electron microscopy and powder X-ray diffraction confirmed the morphological transition from membrane tubes to crystalline branches upon decreasing pH.

## Introduction

In the 17th century, chemical gardens were discovered by J. Glauber.^1^ Thereafter, he inspired many researchers to reveal and understand the underlying chemo-mechanical forces related to such inorganic architectures. He placed metal–salt seeds into water glass solution, which resulted in nonequilibrium self-assembling tubes looking eerily similar to real gardens. The mechanism is well-understood by now: initially a membrane forms around the salt seed, which ruptures due to osmotic pressure gradient across the surface. As buoyancy forces arise, upward growth takes place forming tubes. Over the years, a new scientific field, known as chemobrionics^1,2^ (derived from the Greek word “bruein” meaning to grow), was born based on observing similarities between living or inanimate structures and batch reactions. Some examples of natural chemical gardens include rusts on metals^3^ or hydrothermal vents and chimneys,^4−7^ in which the origin of life may have emerged.^8^ Since the semipermeable membrane allows ion transport in the form of harvestable electric energy,^9−11^ the very first autogenic reactions could have exploited this in prebiotic chemistry.^12^

Among the possible applications, structure design is clearly one of the most spectacular. For example, a wide range of patterns was observed: from regular tubes^13,14^ to popping or budding membranes,^15−17^ through life-imitating biomorphs.^18^ Some of them were considered as hierarchical structures, meaning that the larger-scale components of the structure are constructed from smaller ones.^19,20^ External factors (absence of light,^21^ presence of magnetic field^22^), pH,^23^ or the geometrical arrangement^24^ also play a role in influencing structural evolution. In particular, 2D and 3D flow-injection methods offer a convenient way to control the reaction by applying solutions instead of salt crystals.^15,25−27^ Beyond these liquid-in-liquid systems, reactants also can be provided from gel matrices that are layered directly underneath the other reactant fluid to make thin tubular chemical gardens.^13,20,28,29^

In this study, we investigate the precipitation of calcium phosphate. Previously, calcium chloride solution was dropped into sodium phosphate solution yielding a planar chemical garden^30^ on the solution top or, under microfluidic conditions, their parallel injection resulted in thin calcium phosphate membranes.^31^ Hydroxyapatite (Ca_10_(PO_4_)_6_(OH)_2_, HAP)—the most stable form of all calcium phosphate minerals—has gained a lot of interest due to its similarity to the mineral phase of bones^32,33^ and teeth.^34^ Calcium phosphates are widely used as bioceramics in tissue engineering,^35,36^ for example, as hip-joint endoprostheses.^37^ HAP is an excellent material for dental implants^38^ or for skeletal reconstruction as bioactive cements.^39^ It is still in focus, because controlling the crystallization process is especially challenging.^40,41^ However, we can create structures with appropriate planning in a controlled manner.^42^ For example, pH can significantly influence not only the tubular growth^43^ but also the final morphology.^44,45^ Therefore, it is important to select the starting parameters well ahead.

In this work, we focus on the transition from crystalline phases to membrane formations of calcium phosphate chemical gardens via a 3D flow-driven method at different pHs and flow rates. Microstructure is also characterized, revealing the fundamental differences between morphologies that underlie the distinct mechanical properties of macroscale patterns.

## Experimental Section

Structures were obtained with a 3D flow-injection system:^46^ A plexiglass cuvette (3.1 × 3.1 × 10 cm^3^) served as a reaction vessel, into which 50 mL of calcium chloride solution was poured. Sodium phosphate solution was injected through a Tygon tube (i.d. = 0.76 mm) from below using a peristaltic pump (Ismatic Reglo) at 0.07–1.16 mL min^–1^ volumetric flow rates. The accessible range was bracketed by clogging below the lower limit and constant breaking off at the orifice above the upper limit, both of which also depended on the composition. The bottom plate of the cuvette (*xy* plane) with a hole in its center and the tube ending in a scalp vein needle (i.d. = 0.4 mm) were carefully connected together after both segments had been filled with the appropriate solutions. To avoid any initial clogging, a small amount of solution was removed from the tip of the needle by soaking it up manually with a paper towel. Images in color mode were taken to capture the temporal evolution of the patterns using a digital camera (Unibrain Fire-i 630c) with Vivitar lens. To enhance the contrast of the images, structures were illuminated by LED light and black background was used on the reactor. For characterization of symmetrical balloons, ∼1 mg of methylene blue (Reanal) dye was added to the alkaline solution, and therefore white background was used. All experiments were repeated at least five times, to determine their reproducibility at room temperature 23 ± 2 °C. Samples collected from the cuvette were washed with deionized water and dried before further analysis. A powder X-ray diffractometer (Philips) was used to investigate the composition of the crystalline solids. Scanning electron microscopy (Hitachi S4700, operating at 10 kV accelerating voltage) images complemented the microstructure characterization. We also determined the density of each solution with an Anton Paar DMA 500 digital density meter (see Tables S1 and S2 in the Supporting Information). The density difference (Δρ) between the outer (ρ_Ca^2+^_) and injected (*ρ*_*i*_) solutions was defined as Δρ = ρ_Ca^2+^_ – *ρ*_*i*_.

In all of the experiments, 2.04 M Ca^2+^ solution from analytical grade CaCl_2_·2 H_2_O (VWR) was used as the outer electrolyte. However, injected phosphate solutions were made in different ranges of concentrations: 0.13–0.50 M from Na_3_PO_4_·12H_2_O (technical, VWR), 0.3–0.8 M from Na_2_HPO_4_·12H_2_O (analytical, Reanal), or 0.5 M from NaH_2_PO_4_·2H_2_O (GPR rectapur, VWR). In other cases, where the goal was to make solutions at different pH values, the total concentration of the phosphate ion containing solutions defined as *c*_*T*_ = [PO_4_^3–^] + [HPO_4_^2–^] + [H_2_PO_4_^–^] was set to 0.5 M. The reagents were dissolved in deionized water (Purite RO100).

## Results and Discussion

### Self-Organized Patterns

With careful design, we are able to control structural properties. One way is to vary the initial concentration of the reactant solutions. This affects the saturation and the pH of the medium, as well as the surface charges and the mechanical stability of the chemical structures. Closely related to concentrations, the density difference between the reactant solutions creates a buoyancy force that can support or counteract the growth of the structure. Uncommon to chemical gardens, we used a reversed scenario for the calcium phosphate (CaP) reaction; namely, the sodium phosphate solution with lower density was injected into the calcium chloride solution to support the upward growth by not only injection but also buoyancy. In addition, injection rate is considered as a variable parameter that can lead to different macroscale patterns.

**Figure 1:**
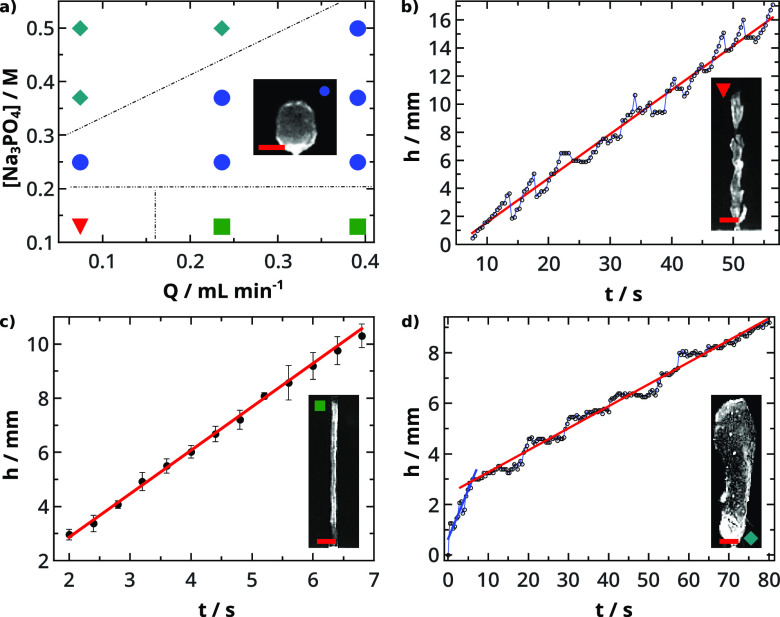
Dynamical phase diagram of CaP chemical gardens at different [Na_3_PO_4_] and flow rates (*Q*) (a). Four structures are observed: worm (⧫), balloon (●), popping (▼), and jetting tube (■). Temporal evolution of height with [Na_3_PO_4_] = 0.13 M and *Q* = 0.07 mL min^–1^ (b), [Na_3_PO_4_] = 0.13 M and *Q* = 0.24 mL min^–1^ (c), and [Na_3_PO_4_] = 0.37 M and *Q* = 0.07 mL min^–1^ (d). Scale bar: 2 mm.

We first present the dynamical phase diagram of the calcium phosphate precipitation, where the concentration of Na_3_PO_4_ was changed along with the flow rate (*Q*) (see Figure 1a). We can distinguish four different growth regimes of thin membrane morphologies. The tube has a small diameter and exhibits axial (vertical) growth, in either intermittent popping or continuous jetting mode. We term wider tubular structures with axial growth as worms. Finally, balloons are hollow structures that grow both axially and radially in time. In the case of the lowest studied Δρ (see Table S1 in the Supporting Information) and injection rate, a popping tube evolves (depicted with a red triangle in Figure 1a). The tip of the membrane tube frequently breaks due to the internal rising pressure from the injected liquid. Growth mechanisms and velocities of three morphologies from the phase diagram are characterized by determining the temporal evolution of their heights. The periodic height fluctuations of popping show an oscillatory growth mechanism (Figure 1b) with an average velocity of 0.315 ± 0.004 mm s^–1^, determined by straight-line fitting. When popping occurs, the tip of the growing structure breaks off due to the growing inner pressure because of the increasing injected volume. Then a new membrane forms, which grows and expands upward until the next detachment. The process repeats itself, resulting in a periodic fluctuation in the height of the structure. Upon increasing the flow rate, jetting tubes form (marked with green squares in Figure 1a). The hollow tube grows continuously from the needle tip, wrapping it around and following the direction of the upward liquid jet. The tube diameter is found to be constant, 0.607 ± 0.028 mm at *Q* = 0.24 mL min^–1^, which increases to 0.728 ± 0.024 mm on increasing the injection rate to *Q* = 0.39 mL min^–1^. For the jetting tube, the height increases linearly with time (see Figure 1c) with a growth rate of 1.606 ± 0.049 mm s^–1^ at *Q* = 0.24 mL min^–1^. Increasing the PO_4_^3–^ concentration sufficiently strengthens the membrane to create greater resistance against the inner pressure. Balloons (depicted with blue circles in Figure 1a) are able to hold injected volumes at different Δρ and *Q* values but less amount than worms (marked with teal diamonds in Figure 1a). While balloons are symmetrical spheres with uneven surfaces in smaller size, worms elongate vertically to extend much higher. Worms grow with a stepping mechanism as shown in Figure 1d. The initial period with *v* = 0.387 ± 0.041 mm s^–1^ growth rate (blue line) represents the balloon formation, while the stepping process proceeds more slowly with *v* = 0.0869 ± 0.0008 mm s^–1^ (red line). During the plateaus, the volume of the structure increases by horizontal expansion at constant height.

**Figure 2:**
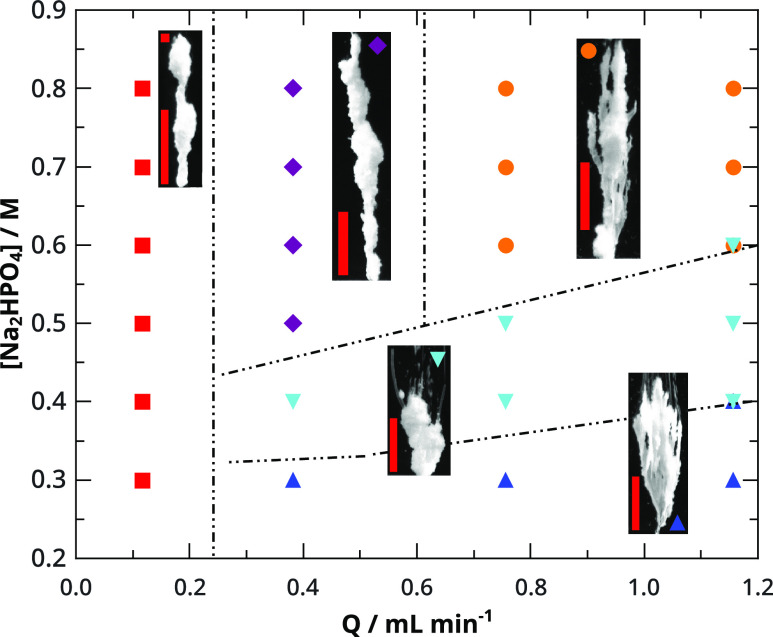
Dynamical phase diagram of CaP gardens at different [Na_2_HPO_4_] and flow rates (*Q*). Five structures are observed: closed tube (■), tube (⧫), branching tube (●), coral-like structure (▲), and a transition structure (▼). Scale bar: 1 cm.

We have also investigated the CaP precipitation reaction where sodium phosphate is replaced by Na_2_HPO_4_. The observed growth regimes are summarized in Figure 2. Applying the more acidic solution results in more rigid, crystalline-based morphologies. At the lowest flow rate, smaller clogging tubes form independently of the concentration (depicted with red squares in Figure 2). Before reaching a maximum height, vertical growth characterizes the tube evolution. Afterward, horizontal precipitation takes place similarly to the formation of a lithium phosphate garden.^47^ At greater concentrations and increased flow rate, taller tubes evolve (depicted with purple diamonds) that reach the upper surface of the outer electrolyte. Further increase of the flow rates produces branches on the tubes (depicted with orange circles). Coral-like structures with hair-like branches are observed at the lowest concentration level at different volume flow rates (blue triangles). In-between (indicated by light blue triangles) thin jetting tubes evolve randomly, on which precipitation appears. From these observations, we can conclude that the increased flow rates create branches, while greater concentrations result in narrower structures with more compact, but smaller, amounts of branches.

### Effect of pH

**Figure 3:**
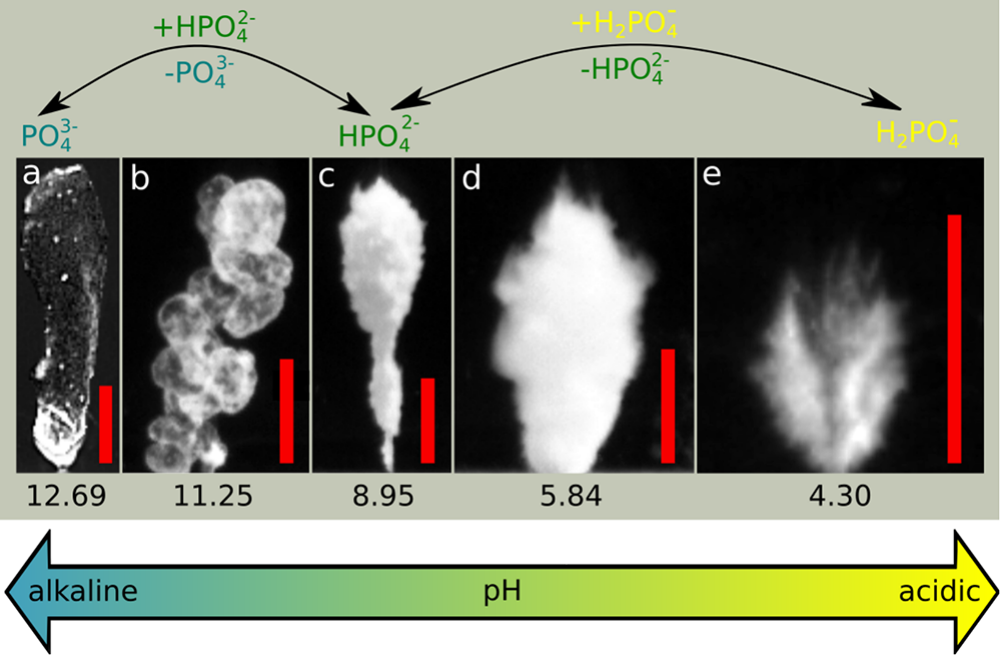
CaP morphologies at different pH values. From alkaline to acidic solutions (a) membrane, (b) membrane mixed with crystalline, (c–e) porous crystalline macroscopic structures observed with decreasing height and increased branching. Scale bar is 0.5 cm in all of the images.

Phosphate ions exist in different protonated states depending on the pH of the solution.^46,48^ In the most alkaline solution, the dominant form is the fully deprotonated PO_4_^3–^, while at pH ≈ 9 HPO_4_^2–^ and at the acidic pH ≈ 4 H_2_PO_4_^–^ are in significant amounts. We have already shown the effect on the morphology when sodium phosphate or disodium hydrogen phosphate solutions are used; however, setting the appropriate pH of the injecting solution results in structures of different sizes and crystallinities (see Figure 3). At the same total phosphate concentration (*c*_*T*_ = 0.5 M) and same flow rate (*Q* = 0.12 mL min^–1^), we have monitored the evolving structures at five different pHs. We can see that worm-like membranes form when pure sodium phosphate solution is injected. At pH = 11.25, an upward growing membrane–crystal composite develops with properties resembling both the membrane and crystalline morphologies. At pH = 8.95, the tubular structure is characterized with a thicker, more crystalline wall. Below pH = 6, the hollow structure is replaced by a porous material with more intensive branching.

**Figure 4:**
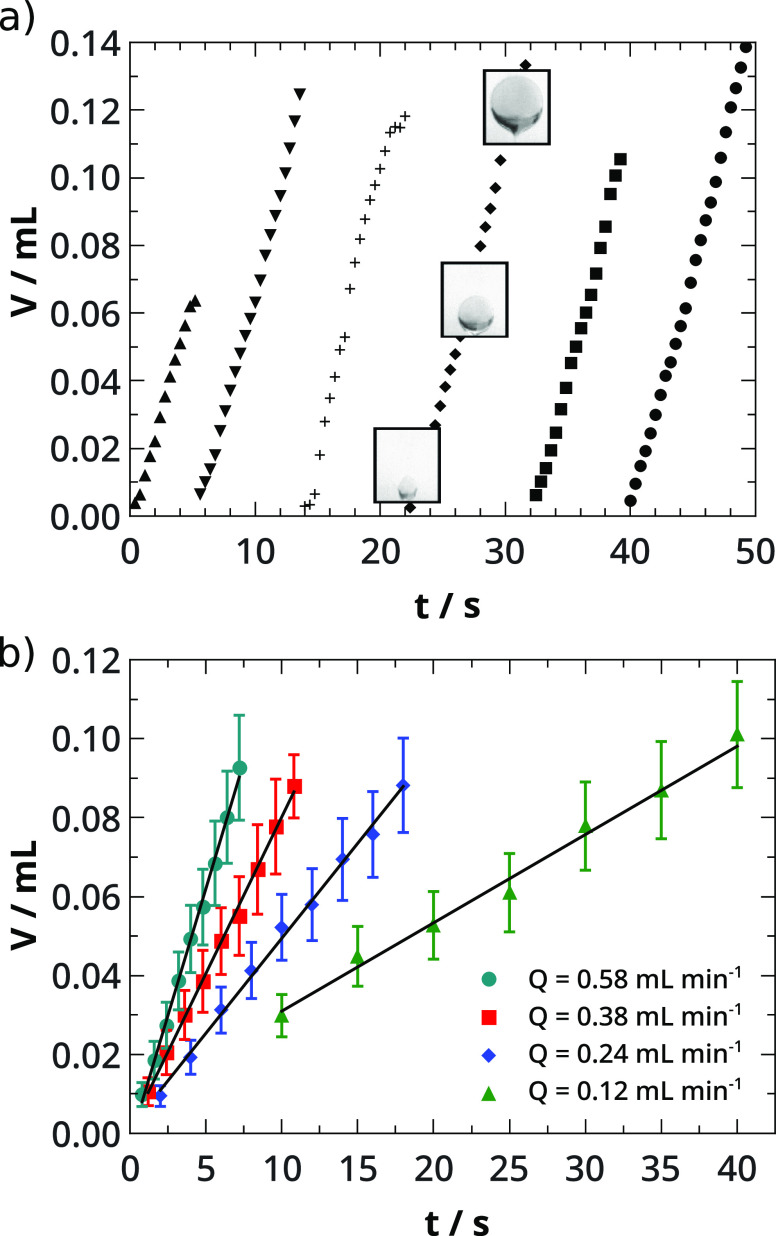
(a) Oscillatory evolution of balloons following the temporal change of their volume at *Q* = 0.58 mL min^–1^. (b) Temporal evolution of balloon volume at pH = 12.46 at different flow rates. Solid lines correspond to straight line fittings.

At pH = 12.46 and 11.82, membrane balloons form, but these are symmetrical and their spherical surface expands evenly compared to the one shown in Figure 1a. At pH = 11.34, irregular balloons evolve. After a certain volume, the balloon detaches from the needle tip due to buoyancy (Figure 4a). This process repeats itself at irregular intervals. In the case of symmetrical balloons, the shape of which can be approximated by a solid of revolution, we are able to analyze their growth at different flow rates at time *t* (Figure 4b) by applying Pappus second centroid theorem^47,49^
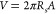
1where *V* corresponds to the volume and *R*_*s*_ to the distance between the axis of rotation and the geometric centroid of the plane with area *A*. The latter is obtained with ImageJ software by taking half of the *xz* cross section of the balloon through its centroid.

The volume grows linearly with time (see Figure 4b) and the slopes, i.e., volume growth rates, match the injection rates within experimental errors. This reveals that precipitate structure captures the injected liquid.

### Microstructure

**Figure 5:**
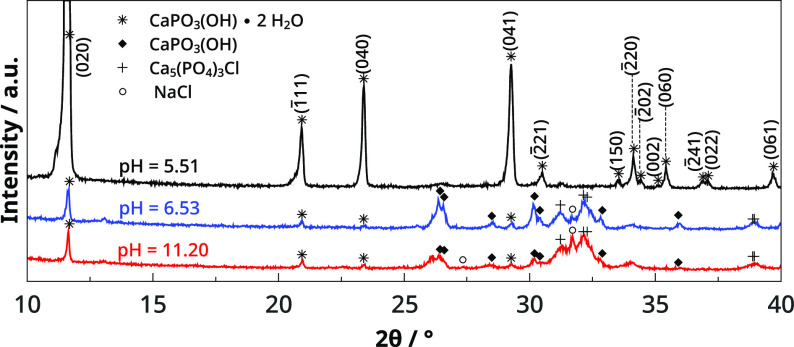
Powder X-ray diffractograms of CaP precipitations.

It has been shown that the pH of the reactants strongly influences structure properties on the macroscale.^44^ Investigating microscopic characteristics may help to understand the different macroscale structures seen in Figure 3. Therefore, samples have been analyzed with powder X-ray diffraction (XRD) at various pH values. The used reference patterns for indexing can be found in Table S3 in the Supporting Information. Three different CaP crystal phases can be identified in the pH range 5.51–11.20, where visible crystals are the dominant macroscale morphology (Figure 5). At pH = 5.51, the diffractogram shows that the crystal composition is only brushite, CaPO_3_(OH)·2H_2_O. It reveals more intense reflections that belong to the (020) family, which suggests specific orientation in these samples. The same composition is detected at pH = 5.84 and pH = 6.08; however, the degree of the specific oriented growth decreases. From pH = 6.53 to pH = 10.95 two other material-specific peaks appear, corresponding to chlorapatite (Ca_5_(PO_4_)_3_Cl) and monetite (CaPO_3_(OH)). In this pH range the specific orientation is not characteristic, but sometimes (020) orientation is over-represented (see Figure S1 in the Supporting Information). As we increase the alkaline character of the anionic reactant to pH = 11.20, the presence of brushite as well as monetite is detected, but the most intense characteristic peak belongs to NaCl as the sample cannot be washed with water without changing its pH. When membrane structures form, we are not able to measure any CaP remains, only sharp NaCl peaks. During sample preparation, the characteristics of the membrane also visibly change, referring to its transformation into white precipitate. However, energy dispersive X-ray analysis (see Figure S2 in Supporting Information) reveals that Ca and P are homogeneously dispersed in the untreated membrane as well as Na and Cl.

**Figure 6:**
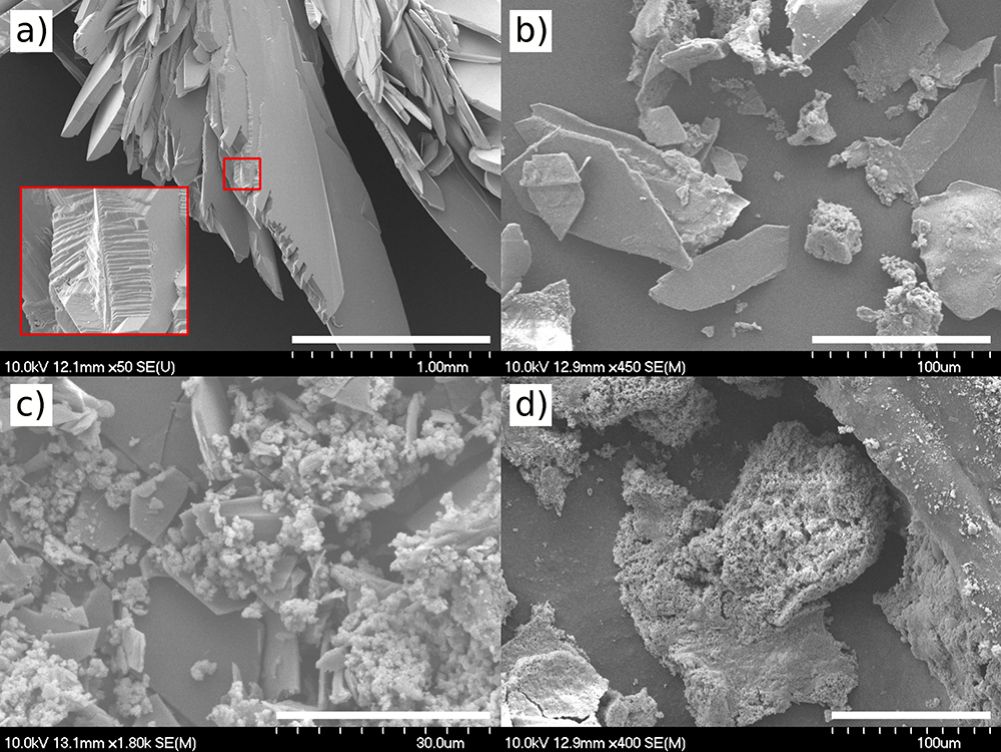
SEM images of a CaP precipitations made with *Q* = 0.12 mL min^–1^ at (a) pH = 4.30, (b) pH = 5.84, (c) pH = 8.95, and (d) pH = 11.25. Scale bars are (a) 1.0 mm, (b and d) 100 μm, and (c) 30 μm.

Scanning electron microscopy (SEM) measurements are also made from different characteristic compositions based on XRD measurements (see Figure 6). Aggregated crystals of brushite are shown in Figure 6a, where hierarchical layers on each other build up the crystal at pH = 4.30. As the phosphate solution is less acidic, large and separate sheets of brushite are formed with fewer sharp edges (Figure 6b) besides other smaller precipitates. This agrees with the XRD data, as more than one crystal form has been detected. As we increase the pH to 8.95 (Figure 6c), the presence of smaller crystals dominates compared to the brushite sheets, which also decrease in size, similarly to previous work.^44^ Lastly, pH = 11.25 is investigated (Figure 6d), where membrane forms on the macroscale. The SEM image reveals its porous structure with a smooth outer surface.

Comparing the XRD and SEM results with the macroscopic structures in Figure 3, we suggest that the macroscale rigidity decreases significantly at lower pH, due to the widely present, but hardly aggregating, bigger planar brushite crystals. The aggregation between crystals increases at higher pH, when smaller monetite and chlorapatite crystals are also formed. In this case, rigid morphologies evolve. It is also important to note that the extension along the horizontal planes are smaller in the case of crystalline structures. The presence of the mixture of the smaller monetite and chlorapatite crystals clogs the pores horizontally and sometimes also vertically, limiting the vertical growth of the structure. At the alkaline pH regimes, thin porous membranes dominate, the rigidity mostly depends on the concentration and, thus, on the rate of the transport processes (Figure 1).

## Conclusions

We have produced 3D calcium phosphate structures, where the pH plays a key role in the morphology both in micro- and macroscale. Various phosphate solutions have been injected into a vessel containing calcium chloride solution. Using tribasic phosphate solution, we can observe popping, in the forms of balloon or worm structures, and jetting tubes having membrane properties evolving via different mechanisms. Their temporal evolution indicates that convection is significant in the structure formation. Tubes of different heights develop from the injections of dihydrogen phosphate ions. Increasing the flow rate results in branching structures, and decreasing the concentration of phosphate solution promotes coral-like structures.

We have also studied how the rigidity of calcium phosphate macrostructures changes along with the microstructure. At very acidic pH, brushite crystals are hierarchically structured. At slightly higher pH, mostly large sheets of brushite crystals grow with barely present aggregation. Therefore, the macroscale structures are less rigid in this pH regime. On increasing the alkaline character of the injected solution, the composition changes as a more crystal phase forms and builds more rigid structures. We suggest that larger amounts of monetite and chlorapatite crystallizing next to the smaller size brushites aggregate together, which clogs the pores of the macroscale structure. We have also observed the porous nature of the alkaline membranes by scanning electron microscopy.

The 3D flow-injection method helps to develop structures in a controlled manner with the careful set of the reactant pH in smaller sizes compared to the industrial scale. Calcium phosphate crystalline phases are always in favor for synthesis of bioceramics, while membranes pave their way to electrochemical applications or in connection with the emergence of life.

## Supporting Information Available

Further details on macro- and microscale characterization: solution density and pH measurements; PXRD and SEM with EDX analyses (PDF)
