# Supplementary material for: From Balloon to Crystalline Structure in the Calcium Phosphate Flow-Driven Chemical Garden
Source: Langmuir. 2023 Mar 27;39(14):5078–83. doi: 10.1021/acs.langmuir.3c00079 (PMC10100542; doi:10.1021/acs.langmuir.3c00079)
Supplement: Supplementary file 1 — la3c00079_si_001.pdf [file la3c00079_si_001.pdf]

Supporting Information: From Balloon to Crystalline Structure in the Calcium Phosphate Flow-Driven Chemical Garden

Réka Zahorán,† Pawan Kumar,† Ágota Deák,‡ Emese Lantos,† Dezső Horváth,¶ and Ágota Tóth*,†

†Department of Physical Chemistry and Materials Science, University of Szeged, Rerrich Béla tér 1., Szeged, H-6720, Hungary.

‡Department of Physical Chemistry and Materials Science, Interdisciplinary Excellence Centre, University of Szeged, Aradi sq. 1, Szeged 6720, Hungary.

¶Department of Applied and Environmental Chemistry, University of Szeged, Rerrich Béla tér 1., Szeged, H-6720, Hungary.

E-mail: atoth@chem.u-szeged.hu

Contents

1 Density and pH measurements **S2**

2 Powder X-ray diffraction (XRD) **S4**

3 Scanning electron microscopy (SEM) and energy-dispersive X-ray spectroscopy (EDX) **S5**

1 Density and pH measurements

Density of the reactants (see Table S1 and S2) was measured with a density meter (Anton Paar DMA-500) at 25 °C. The pH of reactants were measured using a VWR 662-1398 pH electrode controlled by a ThermoOrion model 420 pH meter (see Table S2).

Table S1: Density changes for dynamical phase diagrams

**Composition**	ρ_i **[g cm^{-3}]**	$\Delta\rho = \rho_{Ca^{2+}}$-$\rho_i$ **[g cm^{-3}]**
2.04 M Ca^{2+}	1.1527	-
0.13 M PO_4^{3-}	1.0226	0.1301
0.25 M PO_4^{3-}	1.0453	0.1074
0.37 M PO_4^{3-}	1.0651	0.0876
0.50 M PO_4^{3-}	1.0895	0.0632
0.80 M HPO_4^{3-}	1.0337	0.1190
0.70 M HPO_4^{2-}	1.0643	0.0884
0.60 M HPO_4^{2-}	1.0776	0.0751
0.50 M HPO_4^{2-}	1.0854	0.0673
0.40 M HPO_4^{2-}	1.0909	0.0618
0.30 M HPO_4^{2-}	1.1024	0.0503

Table S2: Density changes for structures developed at different pH

**pH**	**Composition**	ρ_i **[g cm^{-3}]**	$\Delta\rho = \rho_{Ca^{2+}}$-$\rho_i$ **[g cm^{-3}]**
12.69	0.5 M PO_4^{3-}	1.0895	0.0632
12.46	0.45 M PO_4^{3-}+ 0.05 M HPO_4^{2-}	1.0865	0.0662
11.82	0.35 M PO_4^{3-}+ 0.15 M HPO_4^{2-}	1.0819	0.0708
11.34	0.25 M PO_4^{3-}+ 0.25 M HPO_4^{2-}	1.0764	0.0763
11.25	0.225 M PO_4^{3-}+ 0.275 M HPO_4^{2-}	1.0752	0.0775
11.20	0.2 M PO_4^{3-}+ 0.3 M HPO_4^{2-}	1.0739	0.0788
10.95	0.15 M PO_4^{3-}+ 0.35 M HPO_4^{2-}	1.0715	0.0812
10.73	0.1 M PO_4^{3-}+ 0.4 M HPO_4^{2-}	1.0682	0.0845
8.95	0.5 M HPO_4^{2-}	1.0639	0.0888
8.16	0.485 M HPO_4^{2-}+ 0.015 M $H_2PO_4^{-}$	1.0625	0.0902
7.01	0.35 M HPO_4^{2-}+ 0.15 M $H_2PO_4^{-}$	1.0564	0.0963
6.53	0.25 M HPO_4^{2-}+ 0.25 M $H_2PO_4^{-}$	1.0518	0.1009
6.08	0.15 M HPO_4^{2-}+ 0.35 M $H_2PO_4^{-}$	1.0475	0.1052
5.84	0.1 M HPO_4^{2-}+ 0.4 M $H_2PO_4^{-}$	1.0451	0.1076
5.51	0.05 M HPO_4^{2-}+ 0.45 M $H_2PO_4^{-}$	1.0429	0.1098
4.30	0.5 M $H_2PO_4^{-}$	1.0407	0.1120

2 Powder X-ray diffraction (XRD)

The samples were made in the 3D flow-injection system using Q = 1.16 mL min^{-1} to produce sufficient quantity. Samples were then collected, vacuum-filtered, and washed with ∼2 mL deionized water. After that, the samples were left to dry for 5 minutes on 110 °C and then ground in a mortar.

The powder samples were analyzed using a Philips powder X-ray diffractometer (PW 1830 generator, PW 1820 goniometer, Cu Kα: l= 0.1542 nm, 40-50 kV, 30-40 mA, 2θ: 10-70 °, 25.0± 0.5 °C).

Table S3: XRD references

Name	Structure	JCPDS No.
Brushite	$CaPO_3(OH) \cdot 2H_2O$	09-0077
Monetite	$CaPO_3(OH)$	09-0080
Chlorapatite, syn	$Ca_5(PO_4)_2Cl$	33-0271
Halite	NaCl	05-0628

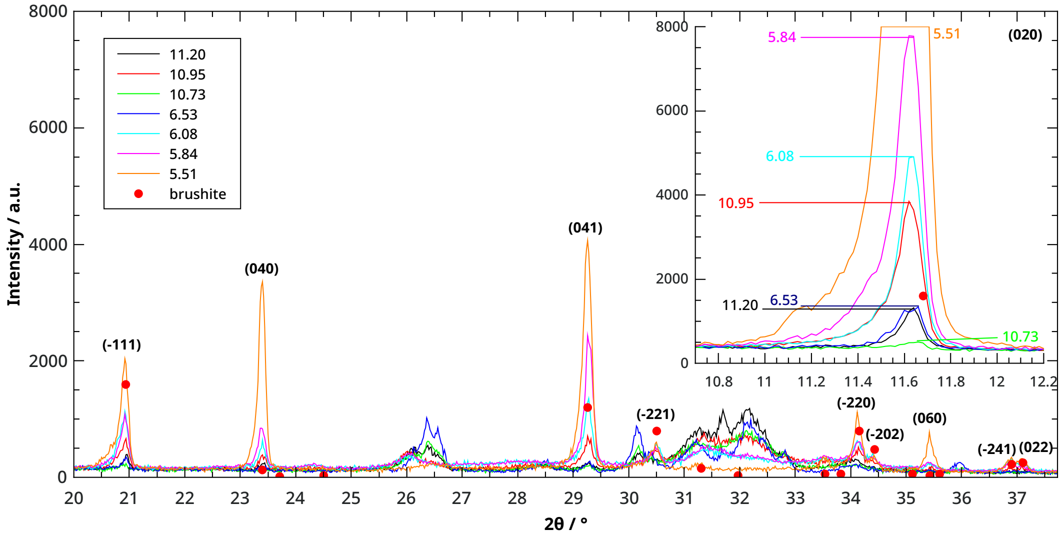

Figure S1: Powder X-ray diffractograms of structures prepared at different pHs. The brushite peaks are indicated with red circles.

3 Scanning electron microscopy (SEM) and energy-dispersive X-ray spectroscopy (EDX)

After taking out the samples carefully from the cuvette, they were rinsed with deionized water and left to dry at room temperature.

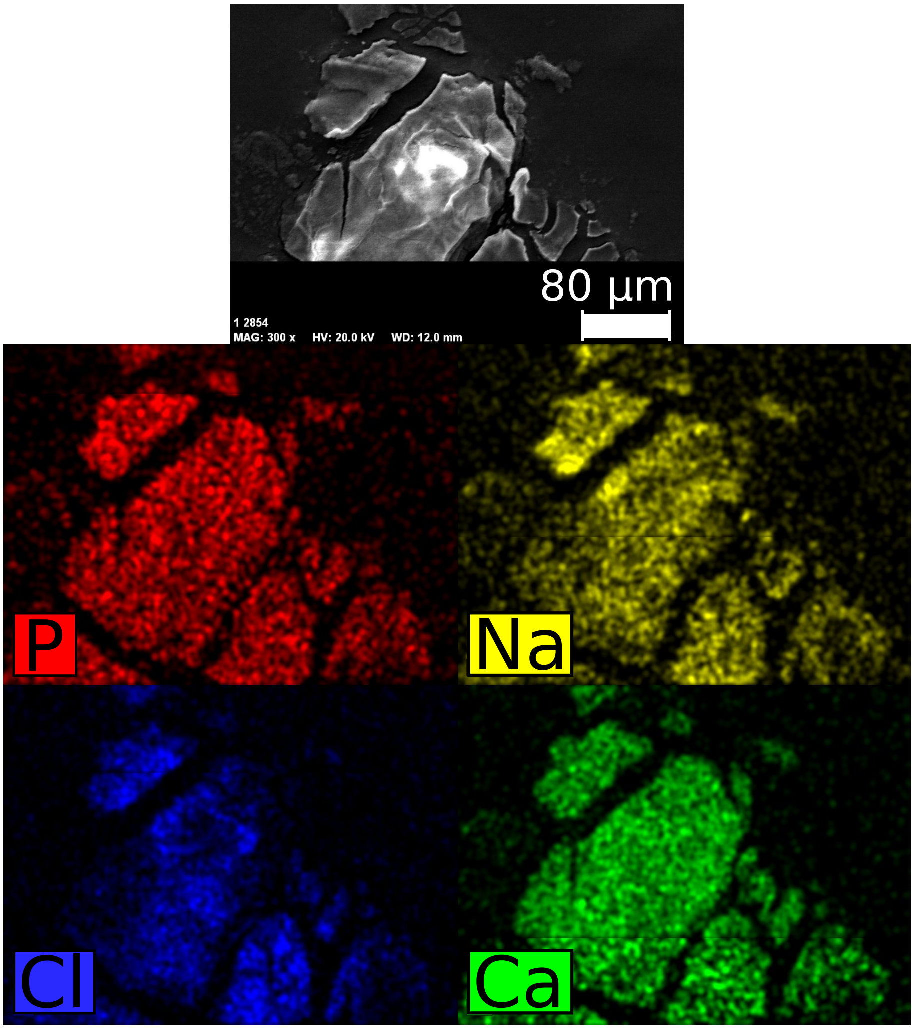

Figure S2: SEM image along with the EDX measurement of the membrane at pH=11.25 and Q = 0.1157 mL min^{-1}. The investigated elements are colored the following way: P (red), Na (yellow), Cl (blue), Ca (green).
